# Angiotensin converting enzyme 1 in the median preoptic nucleus contributes to chronic intermittent hypoxia hypertension

**DOI:** 10.14814/phy2.13277

**Published:** 2017-05-23

**Authors:** Katelynn E. Faulk, T. Prashant Nedungadi, J. Thomas Cunningham

**Affiliations:** ^1^ Institute for Cardiovascular and Metabolic Diseases University of North Texas Health Science Centre at Fort Worth Fort Worth Texas; ^2^ Present address: Office of Science Operations American Heart Association 7272 Greenville Avenue Dallas 76107 Texas

**Keywords:** Angiotensin converting enzyme, central nervous system, Hypertension, sleep apnea

## Abstract

Obstructive sleep apnea is associated with hypertension and cardiovascular disease. Chronic intermittent hypoxia is used to model the arterial hypoxemia seen in sleep apnea patients and is associated with increased sympathetic nerve activity and a sustained diurnal increase in blood pressure. The renin angiotensin system has been associated with hypertension seen in chronic intermittent hypoxia. Angiotensin converting enzyme 1, which cleaves angiotensin I to the active counterpart angiotensin II, is present within the central nervous system and has been shown to be regulated by AP‐1 transcription factors, such as ΔFosB. Our previous study suggested that this transcriptional regulation in the median preoptic nucleus contributes to the sustained blood pressure seen following chronic intermittent hypoxia. Viral mediated delivery of a short hairpin RNA against angiotensin converting enzyme 1 in the median preoptic nucleus was used along with radio‐telemetry measurements of blood pressure to test this hypothesis. FosB immunohistochemistry was utilized in order to assess the effects of angiotensin converting enzyme 1 knockdown on the activity of nuclei downstream from median preoptic nucleus. Angiotensin converting enzyme 1 knockdown within median preoptic nucleus significantly attenuated the sustained hypertension seen in chronic intermittent hypoxia. Angiotensin converting enzyme 1 seems to be partly responsible for regulating downstream regions involved in sympathetic and blood pressure control, such as the paraventricular nucleus and the rostral ventrolateral medulla. The data suggest that angiotensin converting enzyme 1 within median preoptic nucleus plays a critical role in the sustained hypertension seen in chronic intermittent hypoxia.

## Introduction

Obstructive sleep apnea (OSA), which is characterized by interrupted breathing during sleep, is associated with hypertension and other cardiovascular vascular diseases such as heart failure (Nieto et al. 2000; Parati et al. 2007; Dempsey et al. 2010; Javaheri et al. 2017). OSA patients have a sustained hypertension that persists while awake and increased sympathetic nerve activity (SNA) (Carlson et al. 1993; Somers et al. 1995; Smith et al. 1996). This suggests a possible central nervous system (CNS) role in the sustained hypertension and adverse cardiovascular outcomes related to OSA.

Chronic intermittent hypoxia (CIH) is widely used animal model of the arterial hypoxemia seen in OSA patients (Fletcher 2001). CIH not only simulates the arterial hypoxemia from sleep apnea but also produces similar changes in SNA and a sustained diurnal hypertension (Fletcher et al. 1992; Fletcher 2003; Tamisier et al. 2009). Chemoreflex sensitization and other mechanisms have been proposed to account for CIH hypertension (Prabhakar et al. 2007; Dempsey et al. 2010). The model of CIH that we have studied has been shown to produce a neurogenic hypertension based on the effects of ganglionic blockade (Sharpe et al. 2013) Recent studies suggest that the lamina terminalis, which is located along the anterior wall of the third ventricle, contributes to CIH hypertension (Shell et al. 2016). This area consists of two circumventricular organs, the subfornical organ (SFO) and organum vasculosum of the lamina terminalis (OVLT), and the median preoptic nucleus (MnPO) (Brody et al. 1978; Buggy et al. 1978; McKinley et al. 2003; Smith and Ferguson 2010).

Lesions of the ventral lamina terminalis that include the OVLT and ventral MnPO selectively block the development of the sustained, normoxic component of CIH hypertension (Cunningham et al. 2012a). CIH is associated with increased FosB/ΔFosB in the MnPO (Knight et al. 2011) and virally mediated dominant negative inhibition of FosB/ΔFosB in the MnPO blocks CIH hypertension (Cunningham et al. 2012a). FosB/ΔFosB is a member of the AP‐1 transcription factor family that accumulates with chronic or intermittent stimulation of the CNS and is linked to neural adaption (Chen et al. 1997; Herdegen and Leah 1998; McClung et al. 2004). Several genes have been identified in MnPO as possible downstream targets of FosB/ΔFosB that may contribute to CIH hypertension such as the angiotensin converting enzyme 1 (ACE1)(Cunningham et al. 2012a). ACE1 is part of the renin angiotensin system (RAS) which has been shown to contribute to hypertension associated with CIH (Fletcher et al. 1999; Da Silva et al. 2011; Knight et al. 2013; Saxena et al. 2015) and in OSA patients (Kraiczi et al. 2000; Moller et al. 2003). Furthermore, the CNS is known to have its own complete, independent RAS (Grobe et al. 2008; Wright and Harding 2013). ACE1 converts angiotensin I to angiotensin II (ANG II), a central neurotransmitter that affects SNA (Aars and Akre 1968; Grobe et al. 2008). We have previously shown that ACE1 is expressed in MnPO neurons that are activated by CIH and project to the paraventricular nucleus of the hypothalamus an autonomic control region (Faulk et al. 2017). In the MnPO, CIH increases ACE1 mRNA and this increase is blocked by dominant negative inhibition of FosB (Cunningham et al. 2012a). Also CIH increases FosB association with ACE1 mRNA suggesting that FosB regulation of ACE1 in the MnPO may contribute to CIH hypertension (Faulk et al. 2017). In the current study we tested functional contribution of ACE1 to CIH hypertension using viral vectors containing short hairpin RNA (shRNA) against ACE1 injected in the MnPO of rats exposed to CIH. We used a neurotropic Adeno‐Associated Virus serotype (Cunningham et al. 2012a; Walch et al. 2014; Saxena et al. 2015) based on our previous study that showed ACE1 staining in the MnPO is not found in astrocytes and is mainly in neurons (Faulk et al. 2017).

## Methods

All animal procedures were conducted according to National Institutes of Health guidelines and were approved by the Institutional Animal Care and Use Committee at the University of North Texas Health Science Center. Adult male Sprague‐Dawley rats of 6 weeks in age (250–300 g, Charles River Laboratories, Inc., Wilmington, MA, USA) were used for all studies. All rats were on a 12:12 light/dark cycle with a light period of 0700 to 1900 h. Rats were always individually housed and continuously provided with food and water ad libitum in temperature and humidity controlled rooms. All surgical procedures used aseptic technique and rats were treated with carprofen (Rimadyl, 2 mg po) before and after surgery.

### Stereotaxic surgeries

Rats were anesthetized with isoflurane (2–3%), their scalps were shaved and cleaned with betadine, and then they were each placed in a stereotaxic apparatus (David Kopf Instruments, Tujung CA). Their skulls were surgically exposed and leveled between bregma and lambda (Paxinos et al. 1980). Each rat received a microinjection targeted to the MnPO using atlas‐defined coordinates from bregma of 0.0 mm anterior, 0.9 mm lateral and 6.7 mmventral with the injector angled 8 degrees from vertical medial to lateral (Paxinos et al. 1980). After a small burr hole was made into the skull, a 30 gauge injector was advanced to the aforementioned coordinates. Each construct was injected into the MnPO at a volume of 500 *η*L using 5 *μ*L Hamilton syringe (#84851 Hamilton Reno, NV). The injector was left in place for a period of 5 min after the injection for proper absorption of the chosen AAV construct. The hole made in each skull was filled with sterile gel foam followed by closing of their scalps with sterile absorbable suture. The injected viral constructs (GENEDETECT^®^, Auckland, NZ) were used to constitutively express an shRNA against ACE1 with green fluorescent protein (GFP) or a scrambled shRNA with GFP. Each virus was a recombinant AAV1/2 serotype with a CMV promoter and WRE regulatory element. The scrambled control was a random sequence provided by the manufacturer. In our hands, this AAV serotype and promoter combination produces significant expression within 10 to 14 days, is maintained for at least 6 weeks, and is neurotropic (Cunningham et al. 2012a; Walch et al. 2014; Saxena et al. 2015). We chose this approach since our previous study showed that, in the MnPO, ACE1 is expressed almost exclusively in neurons (Faulk et al. 2017). Injection placement was verified by the GFP fluorescence using an Olympus (Olympus BX41) fluorescent microscope or an Arcturus Veritas Microdissection microscope equipped for epifluorescence.

### Radio telemetry transmitter implantation

All rats were implanted with a radio‐telemetry transmitter (TA11PA‐C40 DSI telemetry unit). After recovering from the MnPO microinjections for 1 week, rats were anesthetized with isoflurane (2–3%) anesthesia and their abdomens were shaved and cleaned with betadine and alcohol. Prior to surgery, all instruments and transmitters were sterilized by exposure to pressurized ethylene oxide gas. Using aseptic surgical technique, a midline abdominal incision was made and the nonocclusive catheter was placed in the descending aorta. The transmitter was secured to the abdominal muscle and remained in the abdominal cavity for the duration of the experiment. Sterile prolene suture was used to attach the transmitter to the muscle and sterile vicryl antimicrobial suture was used to close the wound as described previously (Knight et al. 2011). One week of recovery was given before baseline measurements and the CIH protocol.

### CIH protocol

After surgery, the rats were transferred to a room in the animal care facility that contained the CIH apparatus. At the end of the recovery period, cardiovascular parameters ‐ mean arterial blood pressure (MAP), respiratory rate (RR), and heart rate (HR) ‐ were recorded for 5 days of baseline measurements and 7 days of CIH or normoxia. All telemetry signals were sampled for 10 sec every 10 min and combined for hourly averages as previously described (Knight et al. 2011). Rats were randomly assigned to CIH or normoxia treatment groups. CIH exposure was applied for 8 h during the middle of the light phase (0800–1600 h) using a 3 min hypoxia (10% O_2_)/3 min normoxia (21% O_2_) cycle as described previously (Knight et al. 2011). During the remaining time (1600‐0800), the chambers were open to room air. Normoxic controls were placed in the same room but only exposed to room air (21% O_2_) throughout the 7 day protocol. There were four treatment groups: rats injected in the MnPO with AAV‐shSCM exposed to either CIH or normoxia and rats injected in the MnPO with AAV‐shACE1 exposed to CIH or normoxia.

After the 7th day of CIH or normoxia, all rats were injected with inactin (100 mg/kg ip) and sacrificed the following morning 16–18 h after the end of the last CIH cycle (Knight et al. 2011). Some rats were decapitated and their brains were frozen. These brains were used for Laser Capture Microdissection and quantitative RT‐PCR as previously described (Saxena et al. 2015). A separate group of rats were prepared for FosB/ΔFosB immunohistochemistry as previously described (Knight et al. 2011; Cunningham et al. 2012a).

### Laser capture microscopy

**Figure 1:**
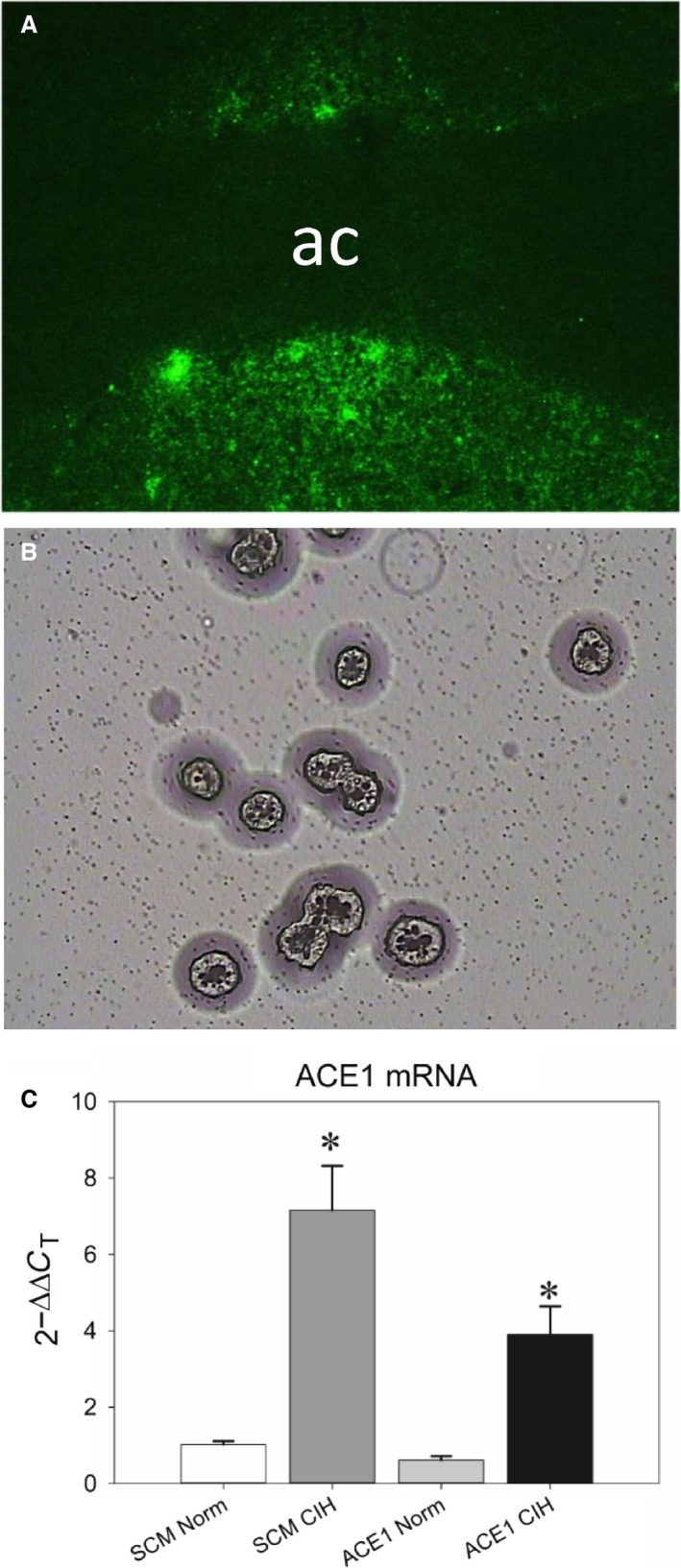
A: Representative digital image of fluorescent green fluorescent protein (GFP) positive cells within the MnPO before LCM (image captured at 100X). B: A brightfield image of GFP cells harvested by LCM and deposited for subsequent analysis (image captured at 200x). C): CIH significantly increased ACE1 mRNA in the MnPO and this effect was significantly attenuated by AAV‐shACE1. * is different from all other groups (Student‐Neuman‐Keuls tests, *P* < 0.05; *n* = 4‐7/group).

Rats were decapitated after anesthetization with inactin (100 mg/kg ip). Brains were removed and immediately frozen in 2‐methylbutane (Sigma‐Aldrich) on dry ice and stored at −80°C. Fresh frozen brains were prepared for laser capture microdissection (LCM) by cutting 10 *μ*m thick serial frozen sections at the level of the MnPO. Six sections were mounted onto PEN membrane coated slides (Catalogue# LCM0522‐ Arcturus Bioscience) and fixed with methanol for 30 sec. An Arcturus Veritas Microdissection instrument (13553‐00, version c), which utilizes an infrared capture laser with an ultraviolet cutting laser, was used to capture 10–15 GFP labeled MnPO cells (Fig. 1A and B). RNA was extracted and purified from each sample using an ArrayPure Nano‐Scale RNA Purification Kit (Epicentre Biotechnol, Madison, WI) (Nedungadi and Cunningham 2014). RNA quality was evaluated using a Nanodrop Spectophotometer (Nanodrop 2000c Spectrophotometer, ThermoScientific, Wilmington, DE) and low 260/280 sample ratios were not used since it was suggestive of wither contamination or very low RNA concentration. The RNA was then amplified to aminoallyl a‐RNA using the TargetAmp 2‐Round Aminoallyl‐aRNA Amplification Kit (epicenter Biotechnol, Madision, WI) as previously described (Carreno et al. 2011; Cunningham et al. 2012b; Nedungadi and Cunningham 2014).

### Quantitative RT‐PCR

Aminoallyl‐aRNA from each sample was reverse‐transcribed into cDNA with a Sensiscript RT Kit (Qiagen Inc., Valencia, CA) as previously described (Saxena et al. 2015). The S18 and ACE1 primers used had the following sequences: Rsp18, forward 5′‐CAGAAGGACGTGAAGGATGG‐3′ and reverse 5′‐CAGTGGTCTTGGTGTGCTGA‐3′; ACE1, forward 5′‐CCCGGAAATACGAAGAATTGC‐3′ and reverse 5′‐GGCTCTCCCCACCTTGTCTC‐3′. Rps1 8 was used for normalization of mRNA expression. PCR reaction mix contained 3 *μ*L of cDNA, 0.6 *μ*L of each primer (forward and reverse), 3.3 *μ*L of RNAse‐free water, and 7.5 *μ*L of iQ SYBR Green Supermix (Bio‐Rad Laboratories Inc., Hercules, CA) for a total reaction volume of 15 *μ*L. PCR reactions were performed with the following protocol in a Bio‐Rad iQTM5 iCycler system (Bio‐Rad Laboratories Inc., Hercules, CA): Denaturation at 95°C for 3 min, 95°C for 10 sec followed by 60°C for 1 min (1 min 10 sec total) repeated for 50 cycles and then 65°C for 5 sec. In each analysis, melt‐curves were generated in order to identify nonspecific products and primer‐dimers.

### Immunohistochemistry

Following the 7 day CIH protocol, rats were given inactin (100 mg/kg ip) and perfused with 0.1 mol/L phosphate buffer saline (PBS, 100–200 mL) followed by 4% paraformaldehyde in 0.1 mol/L phosphate buffer (400–500 mL), as previously described (Cunningham et al. 2012a). Brains were post fixed overnight and dehydrated in 30% sucrose. Each brain was cut into three sets of serial 40 *μ*m coronal sections using a cryostat. The sections were stored in cryoprotectant at −20°C until immunohistochemistry was performed (Knight et al. 2011, 2013). Sections were stained for FosB (Goat polyclonal, sc‐48‐G, Santa Cruz, 1:1000) (Knight et al. 2011, 2013). Tissue processed for DAB staining was incubated with a biotinylated horse anti‐goat IgG (1:200; Vector Laboratories, Burlingame, CA) and treated with an avidin‐peroxidase conjugate from a Vectastain ABC Kit (Vector Laboratories). Tissue was then processed with PBS containing 0.04% 3,3′‐diaminobenzidine hydrochloride and 0.04% nickel ammonium sulfate for 11 min. D*β*H staining was visualized using a Cy3 anti‐mouse (1:250; Jackson ImmunoResearch Inc., West Grove, PA). After the staining procedure, tissue was then mounted to gel‐coated slides, allowed to dry for 1 day. The dried slides were serially dehydrated with ethanol solutions and xylene. Slides were then coverslipped with Permount mounting medium (ThermoScientific, Waltham, MA, USA) and dried for at least 48 h before imaging. Regions of interest were identified according to the atlas of Paxinos and Watson (Paxinos and Watson 1986) as previously described (Knight et al. 2011; Cunningham et al. 2012a). Regions of interest were imaged using an Olympus (Olympus BX41) microscope equipped for epifluorescence and a digital camera (Olympus DP70). ImageJ (1.47v, National Institute of Health, USA) was used to analyze and count labeled cells for each section. Sections were counted by more than one person and the counts were averaged for statistical analysis. FosB/ΔFosB counts were averaged between at least three sections of each brain region. For the paraventricular nucleus of the hypothalamus (PVN, 1.30 mm to 2.12 mm posterior to bregma), we separately analyzed the different parvocellular subnuclei as previously described (Stocker et al. 2005; Saxena et al. 2015) along with an average for the whole nucleus. The nucleus of the solitary tract (NTS) subsections separately analyzed were the commissural/caudal NTS (14.3–14.6 mm posterior to bregma), subpostremal NTS (13.68–14.08 mm posterior to bregma), and rostral NTS (12.6–13.3 posterior to bregma) as previously described (Knight et al. 2011). Three to six sections per rat were also used for the analysis of the RLVM (12.6–13.3 mm posterior to bregma). An average was determined for each region or subregion for each rat that was used for statistical analysis.

### Statistical analysis

Data from baseline the radio telemetry recordings, immunohistochemistry, and qRT‐PCR were analyzed using separate one‐way ANOVAs with Student‐Newman‐Keuls tests for posthoc analysis. Data from the 7 d CIH protocol were analyzed as a change from baseline using separate two‐way repeated measure ANOVA with Student‐Newman‐Keuls tests for posthoc analysis. Rats with injections of AAV‐shACE1 that did not include the MnPO and were exposed to CIH were used to form a separate control group for the analysis of the radio telemetry data. All tests were performed using SigmaPlot (v. 12.0, Systat Software, USA). Statistical significance was set at *P* < 0.05. Data are reported as mean ± SEM.

## Results

### Effects of ACE1 Knockdown in the MnPO and qRT‐PCR

Only rats with GFP centered in the MnPO were used for LCM and qRT‐PCR (Fig. 1A). Rats with injections that were not center on the MnPO were used for LCM and their data were not included in the analysis of the radio telemetry data. The levels of ACE1 mRNA in MnPO were significantly increased by CIH [*F*(3, 19) = 21.39, *P* < 0.001; SNK, *P* < 0.01; Fig. 1C] and this increase was significantly attenuated by the AAV‐shACE1 injections in the MnPO (SNK, *P* < 0.002; Fig. 1C). However, ACE1 message in the rats treated with shACE1 and CIH was still greater than the normoxic controls (SNK, shACE1 +  CIH vs. shSCM.+ Con (*n* = 5), *P* < 0.001; vs. shACE1 +  Con (*n* = 7), *P* < 0.003).

### Effect of ACE1 knockdown on CIH hypertension

**Figure 2:**
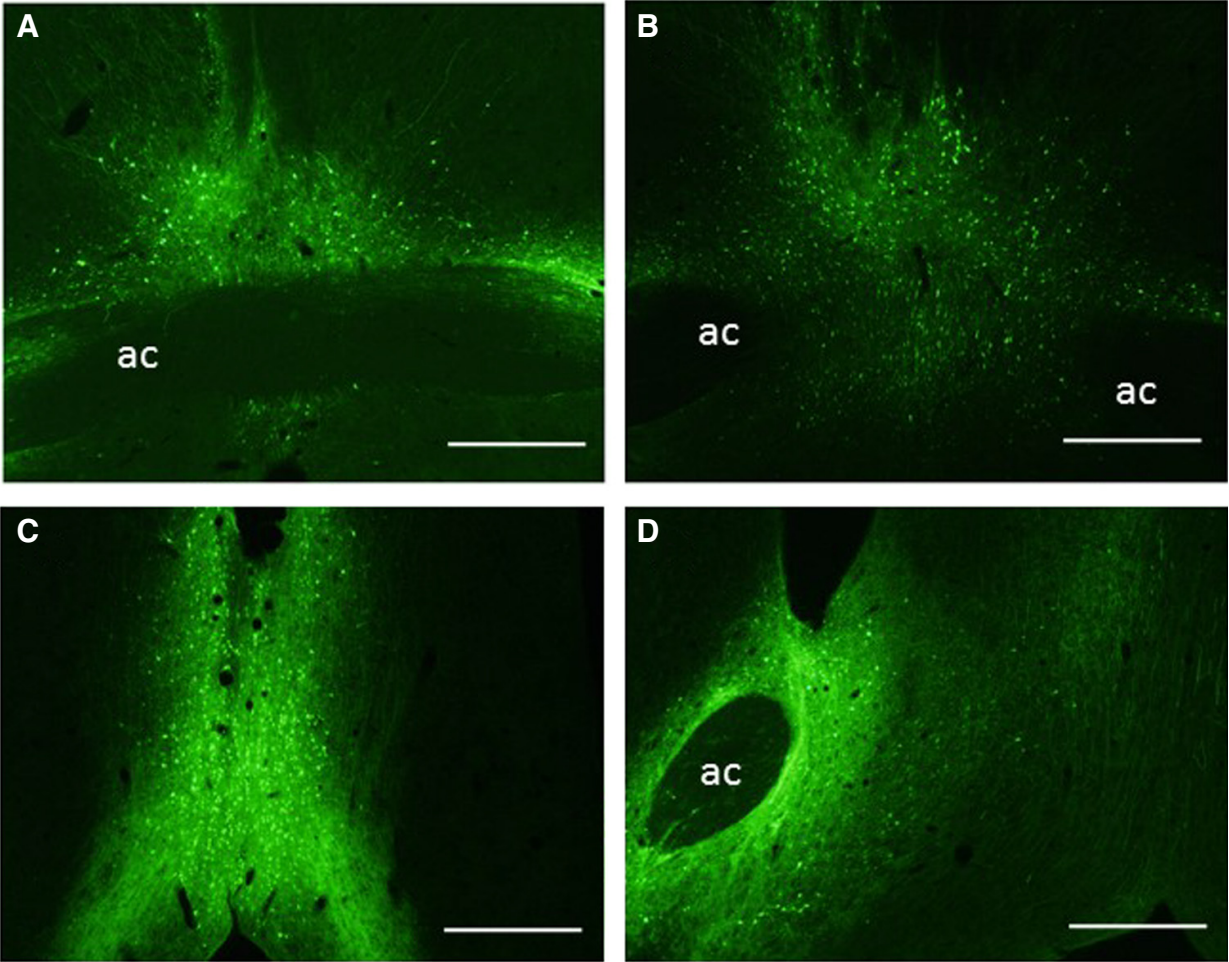
Representative digital images of green fluorescent protein (GFP) labeling in the MnPO from an AAV‐shSCM injection (A) and an AAV‐shACE1 injection (B). Some injections were too anterior and were centered in the diagonal band of Broca (C) or lateral and were located mostly in the bed nucleus of the stria terminalis (D). Scale bars are 500 *μ*m in each image. Abbreviation: ac, anterior commissure.

The injection sites were verified for all brains from the rats used in the analysis of the radio telemetry data. Examples of injections of AAV‐shSCM (Fig. 2A) and AAV‐shACE1 (Fig. 2B) centered on the MnPO are shown in Figure 2. In some rats with injections centered on the MnPO there was spread of the virus into the bed nucleus of the stria terminalis (Fig. 2A). Rats exposed to CIH with injections of AAV‐shACE1 (*n* = 8) that did not include the MnPO were used to form a separate control group. These injection site were either too rostral and were located in the diagonal band or Broca (Fig. 2C) or were off of the midline and were lateral (Fig. 2D).

**Figure 3:**
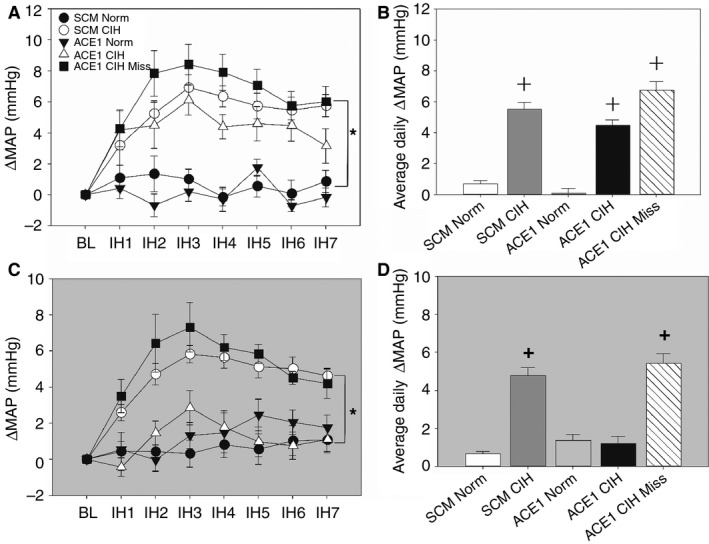
Effects of ACE1 knockdown in the MnPO on changes of mean arterial pressure (MAP) produced by CIH differed during the intermittent hypoxia exposure during the light period (A, white background) and the normoxic dark period (C, gray background) and average daily changes in MAP for the intermittent hypoxia exposure during the light period (B, white background) and normoxic dark period (D, gray background). A: + is SCM and ACE1 CIH group different from both control groups on days 1‐6. On day 7 only the SCM CIH group is significantly different from the Norm groups. B: + is all CIH groups different from both normoxic control groups (*P* < 0.05; *n* = 8‐14/group). C & D: + is SCM CIH and ACE1 CIH Miss different from all other groups (*P* > 0.05; *n* = 8‐14/group).

There were no significant differences in baseline MAP, RR or HR among any of the groups during either the normoxic dark phase and or the light phase from 0800 to 1600 h (Table 1). During the CIH exposures (0800–1600 h), CIH produced increases in MAP in all three treatment groups that were significantly greater than the normoxic control groups [treatment *F*(4, 47) = 25.6, *P* < 0.001; Fig. 3A]. The SCM CIH and ACE1 CIH Miss treatment groups demonstrated significantly greater increases in MAP than the normoxic control groups on all treatment days (SNK, *P* < 0.05; Fig. 3A). The ACE1 CIH group was significantly different from both of the normoxic control groups on all days except for day 7 (SNK, *P* < 0.05: Fig. 3A). The average daily changes in MAP for all of the CIH were significantly greater than both of the normoxic control groups (SNK, *P* < 0.001: Fig. 3B).

**Table 1:** Baseline averages of mean arterial pressure (MAP), heart rate (HR), respiratory rate (RR), and activity recorded for 5 day prior to the start of the intermittent hypoxia protocol in rats that were injected in the MnPO with AAVs containing either scrambled shRNA (SCM) or shRNA targeting ACE1 (ACE1) and later exposed to either 7 days of normoxia (Norm) or intermittent hypoxia (CIH)

	n		MAP (mmHg)	HR (bpm)	RR (bpm)	Activity (cpm)
SCM Norm	14	0800‐1600	96 ± 2.3	330 ± 6	100 ± 1	1.5 ± 0.1
		1800‐0600	03 ± 2.6	385 ± 7	100 ± 1	5.9 ± 0.5
SCM CIH	11	0800‐1600	95 ± 1.2	320 ± 6	97 ± 1	1.0 ± 0.1
		1800‐0600	99 ± 1.1	379 ± 6	99 ± 1	4.5 ± 0.4
ACE1 Norm	11	0800‐1600	94 ± 1.9	319 ± 4	98 ± 1	1.0 ± 0.1
		1800‐0600	100 ± 2.4	386 ± 7	96 ± 1	5.1 ± 0.4
ACE1 CIH	8	0800‐1600	94 ± 2.5	322 ± 4	98 ± 1	1.1 ± 0.2
		1800‐0600	99 ± 2.7	384 ± 5	98 ± 1	4.3 ± 0.4
ACE1 CIH Miss	8	0800‐1600	98 ± 1.9	321 ± 6	100 ± 2	0.9 ± 0.1
		1800‐0600	102 ± 1.8	381 ± 7	101 ± 1	3.9 ± 0.3

Rats with AAV‐shACE1 injections that did not include the MnPO were used to form a separate control group (AC1 CIH Miss). The times in the table correspond to the time of days when the intermittent hypoxia occurred (0800‐1600) or the normoxic dark phase (1800‐0600). There were no significant differences among the groups for any of the variables.

During the normoxic dark phase (1700–0700 h), CIH produced significant increases in MAP in only the SCM CIH and ACE1 CIH miss groups [treatment *F*(4, 47) = 19.9, *P* < 0.001; Fig. 3C]. The daily average changes in MAP of the SCM CIH and the ACE1 CIH Miss group were significantly greater than all of the other groups (SNK, all *P* < 0.02; Fig. 3D). The changes in MAP during the dark phase for the CE1 CIH group was not different from the normoxic control groups (SNK, all *P* > 0.05).

**Figure 4:**
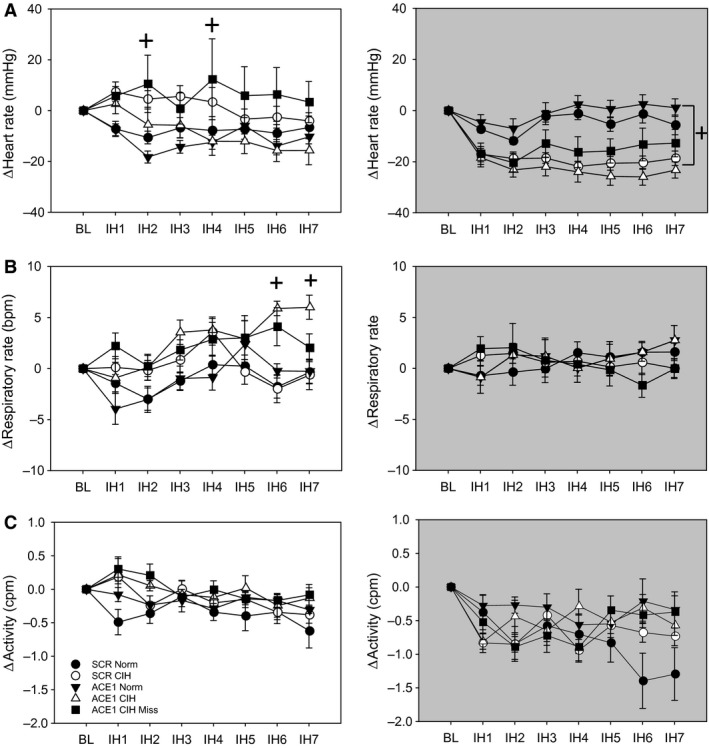
Effects of ACE1 knockdown in the MnPO on changes of heart rate (A), respiratory rate (B), activity (C) produced by CIH during the intermittent hypoxia exposure during the light phase (left column, white backgrounds) and the normoxic dark phase (right column, grey backgrounds). A: + is ACE1 CIH Miss group different from the normoxic control groups during intermittent hypoxia. For the dark period, + is all CIH groups different from normoxic control groups (*P* < 0.05). B: * is significantly different from all other groups during the intermittent hypoxia period (*P* < 0.05). + is both CIH treatment groups significantly different from both bormoxic control groups during the intermittent hypoxia period (*P* < 0.05). C: There were no significant differences in activity among the groups during either time period.

For HR during the intermittent hypoxia treatment period (0800–1600 h), there was a significant interaction between treatment and day [*F*(24, 282) = 2.04, *P* = 0.003] but no main effect of treatment [*F*(4, 47) = 2.46, *P* > 0.05]. The interaction was due to the changes in HR for the ACE1 Miss CIH group being significantly higher than the normoxic control groups on days 2 and 4 (SNK, *P* < 0.05; Fig. 4A). For the dark phase, there was a significant interaction [*F*(24, 282) = 2.15, *P* = 0.002] due to the three CIH treatment groups having significant decreases in HR compared to the normoxic control groups on every treatment day except day 1(Fig. 4A).

For RR during intermittent hypoxia exposure, there was a significant interaction [*F*(24, 282) = 2.51, *P* < 0.001] and a main effect of treatment [*F*(4, 47) = 5.42, *P* < 0.001]. This was due to a trend for the RR to be higher in both of the ACE1 CIH‐treated groups that was significant toward the end of the protocol (SNK, *P* < 0.05; Fig. 4B). During the dark period, there were no differences in RR among the treatment groups [interaction *F*(24, 282) = 0.88, *P* > 0.05; treatment *F*(4, 47) = 0.2, *P* > 0.05; Fig. 4B]. There were no significant treatment effects on activity recorded during IH [*F*(4, 47) = 1.68, *P* < 0.05; Fig. 4C] or the dark phase [*F*(4, 47) = 1.18, *P* < 0.05; Fig. 4C].

### Effects of MnPO ACE1 Knockdown on CIH‐induced FosB Staining

#### Effect of ACE1 knockdown on FosB/ΔFosB staining in MnPO

**Figure 5:**
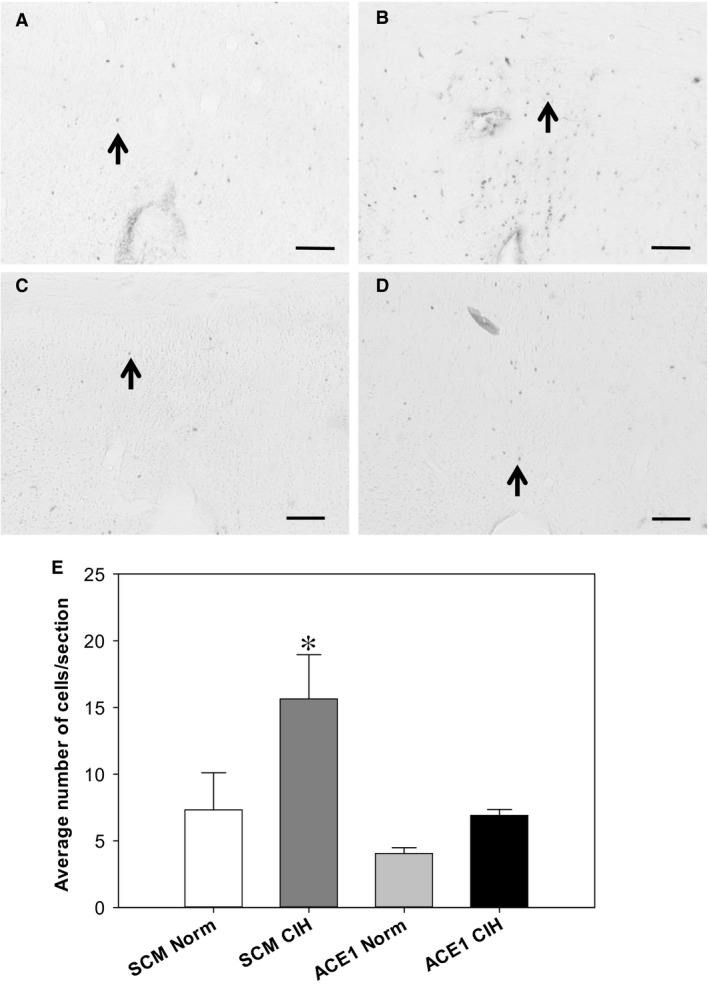
Effects of shACE1 knockdown on FosB staining in the MnPO. Representative images for each treatment group ‐ A: SCM Norm, B: SCM CIH, C: ACE1 Norm and D: ACE1 CIH. E: Summary data for FosB staining in the MnPO (*n* = 6/group). * is significantly different from all other groups (*P* < 0.05). Scale bars are 100 *μ*m. Arrows indicate FosB positive cells.

FosB/ΔFosB staining within the MnPO was significantly increased in the AAV‐shSCM CIH treatment group as compared to all other groups [*F*(3, 20) = 5.19, *P* = 0.011; SNK, all *P* < 0.05; Fig. 5]. There were no other differences among the groups (*P* < 0.05; Fig. 5).

#### Effect of ACE1 Knockdown on FosB/ΔFosB staining in PVN

**Figure 6:**
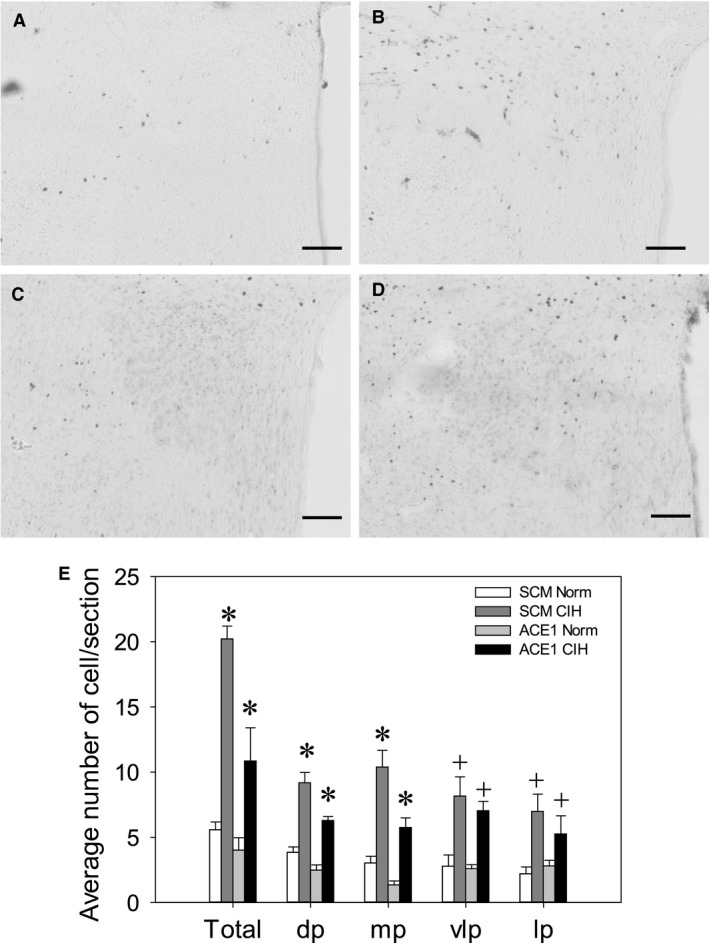
Effects of shACE1 knockdown on FosB staining in the PVN. Representative images for each treatment group ‐ A: SCM Norm, B: SCM CIH, C: ACE1 Norm and D: ACE1 CIH. E: Summary data for FosB staining in the total PVN (Total), dorsal parvocellular region (dp), medial parvocellular (mp), ventrolateral parvocellular and lateral parvocellular regions (*n* = 6/group). * is significantly different from all other groups (*P* < 0.05). + is both CIH groups different from both CON groups (*P* < 0.05) Scale bars are 100 *μ*m.

FosB/ΔFosB staining in the PVN was significantly increased in both the shSCM and shACE1 CIH groups [*F*(3, 20) = 24.5, *P* < 0.001; Fig. 6). The numbers of FosB/ΔFosB positive cells within the PVN of the shSCM CIH groups were significantly increased compared to all of the other groups (SNK, all *P* < 0.001; Fig. 6). The shACE1 CIH treatment group was significantly increased as compared to both normoxic controls groups (SNK, shACE1 CIH vs. shACE1 Con, *P* = 0.01; vs. shSCM Con, *P* = 0.02). ACE1 knockdown in the MnPO significantly attenuated FosB/ΔFosB staining overall in the PVN. Similar results were observed in the dorsal parvocellular [*F*(3, 20) = 31.8, *P* < 0.001; SNK all *P* < 0.01; Fig. 6) and medial parvocellular regions of the PVN [*F*(3, 20) = 24.2, *P* < 0.01; SNK all *P* < 0.05; Fig. 6).

#### Effect of ACE1 Knockdown on FosB/ΔFosB staining in Hindbrain

**Figure 7:**
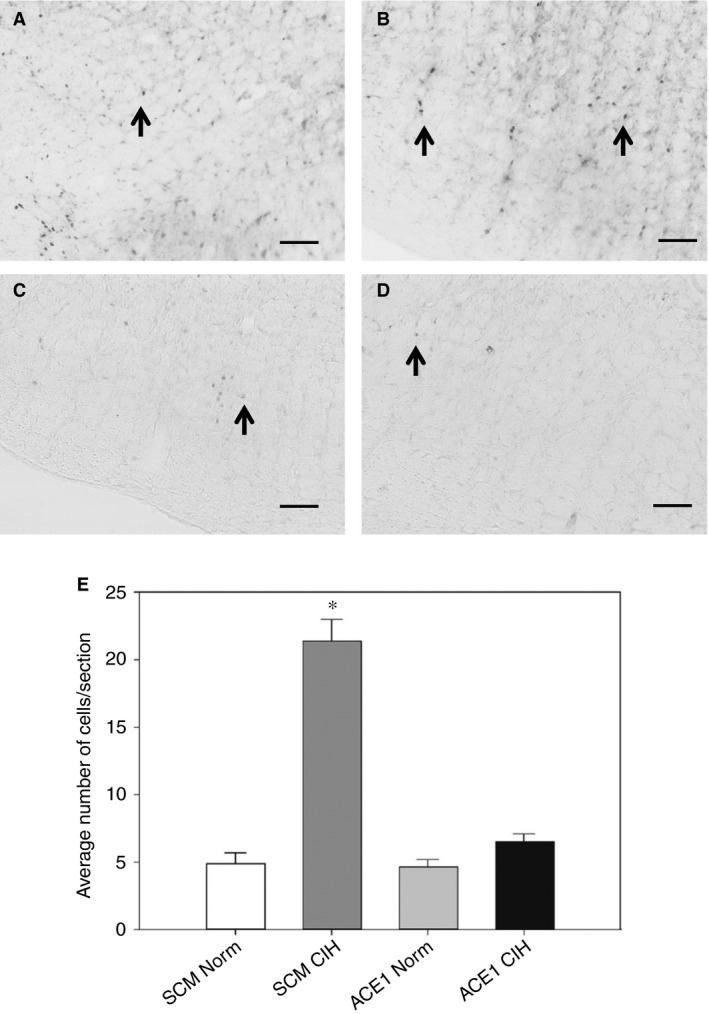
Effects of shACE1 knockdown on FosB staining in the RVLM. Representative images for each treatment group ‐ A: SCM Norm, B: SCM CIH, C: ACE1 Norm and D: ACE1 CIH. E: Summary data for FosB staining in the RVLM (*n* = 6‐8/group). * is significantly different from all other groups (*P* < 0.05). Scale bars are 100 *μ*m. Arrows indicate FosB positive cells.

**Figure 8:**
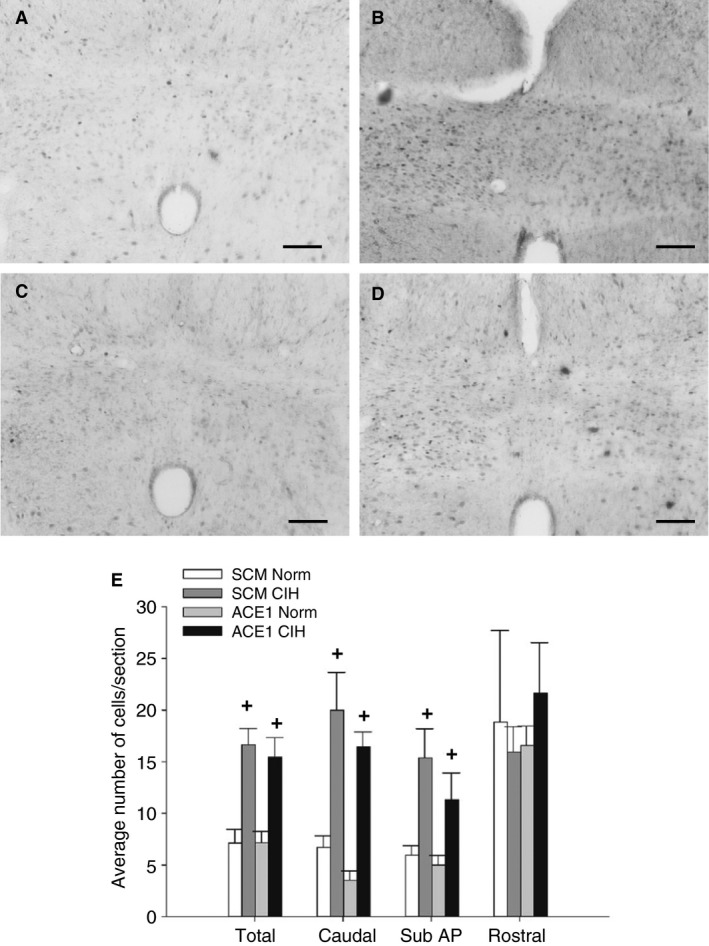
Effects of shACE1 knockdown on FosB staining in the total NTS. Representative images for each treatment group ‐ A: SCM Norm, B: SCM CIH, C: ACE1 Norm and D: ACE1 CIH. E: Summary data for FosB staining in the NTS (*n* = 6‐8/group). + is both CIH groups significantly different from both CON groups (*P* < 0.05). Scale bars are 100 *μ*m.

In rats injected in the MnPO with AAV‐shSCM and exposed to CIH, there was a significant increase in FosB/ΔFosB staining within the RVLM as compared to all treatment groups [*F*(2, 23) = 70.85, *P*, 0.001; SNK, all *P* < 0.001; Fig. 7). In contrast, ACE1 knockdown in the MnPO did not significantly influence FosB/ΔFosB staining in the NTS (Fig. 8). The total number of FosB/ΔFosB positive cells within the entire NTS significantly increased in both CIH groups whether AAV‐shSCM or AAV‐shACE1 as compared with both normoxic controls [*F*(3, 16) = 12.13, *P* < 0.001; SNK all *P* < 0.01; Fig. 8]. Similar increases in both CIH groups were seen in the subpostremal [*F*(3, 16) = 5.74, *P* = 0.007, SNK all *P* < 0.05) and caudal subdivisions of the NTS which showed a significant increase in CIH‐treated groups as compared with the normoxic counterparts (Fig. 8). However there were no significant differences among the treatment group within the rostral subdivision of the NTS (Fig. 8).

## Discussion

The results of this study support the hypothesis that ACE1 is a FosB regulated gene in the MnPO that contributes to the sustained, normoxic component of CIH hypertension. Injections of AAV‐shACE1 in the MnPO blocked the increase in ACE1 message associated with CIH and prevented the increase in MAP during the normoxic dark phase of the CIH protocol. They did not prevent increases in blood pressure during the CIH exposure period from 0800 to 1600 h. MnPO ACE1 knockdown also did not consistently influence HR or RR during the intermittent hypoxia period or during the normoxic dark period.

These results are similar to the effects of lesions of the anteroventral region of the third ventricle and virally mediated dominant negative inhibition FosB in the MnPO (Cunningham et al. 2012a). It has also recently been shown that CIH increases the association of FosB with ACE1 mRNA in the MnPO (Faulk et al. 2017). Further, we showed that, in the MnPO, ACE1 is expressed in neurons that project to the PVN and are FosB positive after CIH while it is not expressed in astrocytes (Faulk et al. 2017). Since FosB inhibition and ACE1 knockdown both selectively blocked the sustained component of CIH hypertension that occurs during normoxic, it is possible that transcriptional regulation of ACE1 is one of the mechanisms necessary for CIH hypertension by which FosB contributes to neural adaptions at the level of the MnPO. Our results provide, possibly for the first time, an activity dependent mechanism in the MnPO that supports CIH hypertension through ACE1. This mechanism may be relevant to other models of hypertension given the role of the anteroventral region of the third ventricle, which includes the ventral MnPO, in neurogenic hypertension (4, 5).

ACE1 knockdown in the MnPO also produced regionally specific effects on FosB/ΔFosB staining. In the MnPO, ACE1 knockdown blocked the increase in FosB/ΔFosB staining associated with CIH. These data suggest that ACE1 within the MnPO not only is regulated by FosB/ΔFosB as indicated by our previous studies (Cunningham et al. 2012a; Faulk et al. 2017) but that ACE1 may support increased activity in the MnPO that is necessary for increased FosB expression during CIH. In the MnPO, this type of interaction between FosB and the local RAS could create a feed forward loop that helps sustain CIH hypertension in the absence of hypoxia. This interaction would likely also depend on angiotensin related input from the SFO (Saxena et al. 2015). Alternatively, ACE1 is involved in the metabolism of several neuropeptides including bradykinin that could also be involved in CIH hypertension at the level of the MnPO (Masuyer et al. 2014).

A decrease in FosB/ΔFosB staining in the PVN was noted after knockdown of ACE1 in the MnPO. The PVN has been shown to regulate sympathetic outflow (Barman 1990; Guyenet 2006) and changes in sympathetic response to hypertonic saline produced by CIH are dependent on the PVN (Sharpe et al. 2013). The effects on FosB/ΔFosB staining that were observed in the current study occurred primarily in the dorsal and medial parvocellular regions. The PVN neurons in the dorsal parvocellular region are known to project to the RVLM, the intermediolateral column of the spinal cord, and the dorsal vagal complex (Swanson and Kuypers 1980). This portion of the PVN is innervated by the MnPO (McKinley et al. 2015). A reduction in FosB/ΔFosB staining in this part of the PVN associated with ACE1 knockdown in the MnPO is consistent with our previous data demonstrating that ACE1 positive cells in the MnPO project to the PVN (Faulk et al. 2017). It is possible that the increase in MnPO ACE1 associated CIH could increase ANG II signaling, or another peptide metabolized by ACE1, to the PVN. ANG II has been shown to activate the PVN influencing SNA as well as blood pressure (Lenkei et al. 1995; Ferguson and Washburn 1998). Two different mechanisms involving the PVN have been shown to contribute to CIH hypertension. One involves a vasopressin projection to the RVLM which would contribute to increased SNA and hypertension in CIH (Kc et al. 2010; Prabha et al. 2011). The other mechanism involves an oxytocin projection to the dorsal vagal complex that when activated ameliorates hypertension in rats exposed to CIH with hypercapnia (Jameson et al. 2016). The contributions of the PVN to autonomic changes associated with CIH are complex and will require additional investigation.

ACE1 knockdown in the MnPO also significantly reduced CIH‐induced FosB staining in the medial parvocellular subregion of the PVN which contains neuroendocrine neurons that regulate the adenohypophysis (Simmons and Swanson 2009). CIH has been shown to sensitize that HPA axis resulting in potentiated responses to heterotypic stressors (Ma et al. 2008). It could be that ACE1 in the MnPO contributes to this neuroendocrine effect but this was not tested in this study. We did not observe significant effects of ACE1 knockdown on CIH‐induced FosB staining in other subregions of the PVN. This result could be related to studies demonstrating a chemoreceptor pathway to the PVN (Reddy et al. 2005; King et al. 2012; Bathina et al. 2013). The lack of an effect of the MnPO knockdown on FosB/ΔFosB staining in the ventrolateral and lateral parvocellular PVN could be due to chemoreceptor stimulation or hypoxia related to CIH.

ACE1 knockdown within the MnPO significantly decreased FosB/ΔFosB staining in the RVLM associated with CIH. This region of the medulla contains sympathetic premotor neurons that regulate blood pressure (Guyenet 2006). Decreased FosB/ΔFosB staining in the RVLM is consistent with the effects of MnPO ACE1 knockdown on CIH hypertension. CIH‐induced FosB/ΔFosB staining in the PVN and RVLM appear to be dependent on ACE1 in the MnPO. This suggests that these two regions are regulated by ACE1 within the MnPO as part of the mechanism for the sustained component of CIH hypertension. The MnPO projects directly to the PVN (McKinley et al. 2015) which in turn projects to the RVLM as well and to sympathetic preganglionic neurons in the intermediolateral column of the spinal cord (Swanson and Sawchenko 1980). It could be that during CIH the MnPO helps to regulate the activity of the RVLM through these or other pathways.

In contrast to the PVN and RVLM, ACE1 knockdown within the MnPO did not significantly alter the FosB/ΔFosB staining in the NTS. This was similar to the results of our previous study using ΔFosB dominant negative inhibition in the MnPO (Cunningham et al. 2012a). The lack of an effect on FosB/ΔFosB staining within the NTS by knockdown of ACE1 in the MnPO suggests that MnPO ACE1 does not contribute to this effect of CIH and that the chemoreflex mechanism that alters blood pressure is still intact. The respiration rate significantly increasing on certain days during CIH (0800–1600 h) in both CIH‐treated groups further suggests that this mechanism does not alter chemoreflex function during CIH (Prabhakar et al. 2007). Furthermore, chronic activation of the NTS due to chemoreceptor stimulation could have contributed to lack of an effect of the knockdown on FosB/ΔFosB staining in some regions of the PVN as suggested above. These results could also suggest that the contribution of the MnPO and the SFO to CIH hypertension showed in this and our earlier studies (Cunningham et al. 2012a; Saxena et al. 2015; Faulk et al. 2017) is chemoreceptor independent. This could mean that manipulations targeting the lamina terminalis, which selectively interfere with the neural adaptations necessary for the maintenance of CIH hypertension during normoxia, would compromise chemoreceptor regulation of blood gases.

### Perspectives

The sustained hypertension associated with OSA put these patients at risk for serious cardiovascular sequela that can adversely affect their mortality (Nieto et al. 2000; Parati et al. 2007; Dempsey et al. 2010; Javaheri et al. 2017). Since there is a significant prevalence of OSA and the incidence is steadily rising, the underlying mechanisms that are responsible for the sustained hypertension need to be elucidated. This study provides new information about the role of ACE1 within the MnPO in the hypertension associated with CIH and the potential use of ACE blockers that cross the blood‐brain‐barrier in treating hypertension of mild to moderate OSA patients. More studies are needed to understand the mechanisms by which ACE1 contributes to CIH hypertension.

## Conflict(s) of Interest

None.
